# Dexamethasone attenuates echinococcosis-induced allergic reactions via regulatory T cells in mice

**DOI:** 10.1186/s12865-016-0141-4

**Published:** 2016-03-11

**Authors:** Qin Zhang, Jianrong Ye, Hong Zheng

**Affiliations:** Department of Anaesthesiology, the First Affiliated Hospital of Xinjiang Medical University, Urumqi, 830011 China

**Keywords:** Allergic reaction, Cystic Echinococcosis, Dexamethasone, Regulatory T cells

## Abstract

**Background:**

Cystic echinococcosis (CE), caused by infection with *Echinococcus granulosus* larvae, is a potentially life-threatening disease in humans. Anaphylactic shock caused by CE is very dangerous, and is highly prevalent during surgery. Dexamethasone (DEX) is used clinically before operations to prevent allergic reactions; Regulatory T cells (Treg cells) are believed to be associated with negative immune response, which play an important role in alleviating allergic reactions. However, the association of Treg cells with DEX remains unknown.

**Methods:**

In this study, C57BL/6 mice were divided into uninfected group, untreated group and DEX group which were inoculated with protoscoleces from *E. granulosus* and sensitized using a cyst fluid suspension to induce anaphylactic shock. In addition, the mice in DEX group were treated with 10 mg/kg DEX by intraperitoneal injection 30 min before being sensitized.

**Results:**

It was found that 93.75 % of all sensitized mice experienced allergic symptoms. The levels of IgE, IgE/IgG, and IgE/IgG1 were significantly higher in both untreated group and DEX group. The proportion of CD4 + CD25 + FOXP3 + Treg cells relative to CD4+ Treg cells, and the levels of interleukin-10 (IL-10) and tumor growth factor-β (TGF-β1) were significantly higher in DEX group. The level of IL-13 was significantly higher in the sensitized mice than in the other groups. These cells may play a key role in alleviating the immune response in CE-induced anaphylactic shock.

**Conclusions:**

The protective effect of DEX may be due to Treg cell upregulating IL-10 and TGF-β1 levels, and inhibiting helper T cell 2 cytokines.

## Background

Cystic echinococcosis (CE), or hydatid disease, is a widespread chronic endemic helminthic disease, commonly caused by infection with metacestodes (larval stages) of the tapeworm *Echinococcus granulosus* (*E. granulosus*) [1–3]. At beginning, few clinical symptoms are noticed. Until hydatid cysts of *E. granulosus* begins to grow big, oppression symptom such as abdominal distension and pain may happen. Sometimes, the hydatid cyst rupture spontaneous or provoked by external pressure in accident which will produce lethal effects [4]. The most recent data shows that the intra-operative incidence of anaphylactic shock is approximately 2 % in CE patients in China [5].

CE-induced anaphylactic shock is a type I allergy synergized by endotoxin shock. Patients usually have severe clinical symptoms, long-lasting hypotension, poor response to vasoactive drugs, and high mortality [6], which are different with the traditional type I allergy. Patient needs long-time positive vasoactive agent infusion, which leads to more adverse effects. Thus, elucidating the mechanism underlying echinococcosis-induced sensitization is crucial for the development of effective prevention and treatment protocols for these patients. Previous studies [7–10] find that, the Th2 response is the prevalent response in subjects with atopy and some allergic disease. With the growth of parasite, helper T cell 2 (Th2) cell responses and some cytokine profiles have more benefit effect to protect host against helminthic infections. On the contrary, the helper T cell 1 (Th1) responses are more likely to occur secondary to specific microbiologic insult. Recent research [11] has shown that T cells are activated after CE infection, but the significant increase in the level of Treg cells could inhibit T cell response to *E. granulosus* antigens, suggesting that these cells may play a key role in the downregulation of the immune response in CE infection. This indicates that Treg cells play an important role in parasite persistence during chronic echinococcosis to maintain the immune stable status.

Immune system is a complex regulatory net. Allergy, tolerance, and active suppression may not be independent events, but rather involve similar mechanisms. In some special condition, Treg cells may redirect an inappropriate immune response against allergens. Original tolerance status may change, allergy happen. Induction of allergen-specific Treg cells seems essential for maintaining a healthy immune response towards allergens [12, 13]. In CE infection, the parasites and the host co-exist in a state of balance for a long time, but the balance is broken when hydatid cysts rupture, causing a severe allergic reaction. Currently, there are no suitable methods for preventing the occurrence of anaphylactic shock caused by CE. Dexamethasone (DEX) is used clinically before operations to prevent allergic reactions. DEX has a broad-spectrum anti-inflammatory allergy effect [14], mainly depending on reducing inflammation factors, but does DEX really work, and if it does, what is its mechanism of action? Relatively, little information is available regarding host immune responses following CE-induced anaphylactic reaction, and therefore little information is available regarding the nature of these responses. Illustrating the characteristics of these immune cells will help the development of new strategies for the treatment and prevention of anaphylactic reactions.

In this study, we investigated several immune cell populations in mice that were either healthy or challenged with *E. granulosus* after infection. The objective is to clarify the changes in the host immune responses during allergic reaction status, and determine the effectiveness and underlying mechanism of DEX in the treatment of CE-induced anaphylactic reaction.

## Methods

### Animals

Forty female C57BL/6 mice weighing 22–25 g (aged 5–7 weeks) were provided by the Animal Center of the First Affiliated Hospital of Xinjiang Medical University. Standard guidelines for laboratory animal care (Institute of Laboratory Animal Resources, Commission on Life Sciences, National Research Council, 1996) were followed. All mice were provided with water and standard chow pellets ad libitum for 2 weeks prior to the start of the experiment. This study was carried out in strict accordance with the recommendations in the Guide for the Care and Use of Laboratory Animals of the National Institutes of Health. The study protocol was reviewed and approved by the Institutional Animal Ethics Committee of Xinjiang Medical University.

### Preparation of antigen and protoscolex

According to the previous study [15], sheep-derived *E. granulosus* cyst fluid (sheep hydatid fluid, SHF) and protoscolex were separated from the livers of sheep infected with *E. granulosus* under aseptic conditions. After precipitation, the supernatant of cyst fluid was obtained. The protoscolexes in the precipitate were washed with normal saline containing 1000 U/ml penicillin and streptomycin for 3–4 times. A dye exclusion test [16] confirmed that the activity of protoscolexes was > 90 %. After resuspension of protoscolexes with normal saline (10000/mL), the supernatant was used as a sensitizing antigen. Bio-Rad protein assay (Bio-Rad, Richmond, CA, USA) determined that the total protein content was 2841.331 μg/ml.

The level of endotoxin contamination in SHF was determined to be 0.03 endotoxin unit (EU)/ml by quantitative chromogenic Limulus amebocyte lysate assay (BioWhittaker Inc., MD, USA). The lipopolysaccharide (LPS) was tested using a commercially available chromogenic LAL end-point assay kit (Cambrex Inc., IA, USA). The level of LPS in SHF was below 1.2 EU/mg protein. The supernatant was stored at −80 °C, and then at 4 °C for 24 h before sensitization.

### Animal grouping and *E. granulosus* infection model establishing

Forty female C57BL/6 (aged 6–8 weeks) mice were randomly divided into uninfected group (n = 8) and infected groups (n = 32). In the infected groups, all the mice were injected percutaneously with 2000 protoscoleces from *E. granulosus*. After 3 months, the presence of cysts in the peritoneal cavity was confirmed by B-scan ultrasonography. Sixteen CE-infected mice were randomized into untreated group and DEX group (n = 8) and the sensitization was induced by intraperitoneally injecting SHF with dose of 0.1 ml/10 g [17, 18]. The uninfected group received phosphate-buffered saline instead of SHF antigens. The DEX group was treated with 10 mg/kg DEX (Sigma-Aldrich Corp., MO, USA) by intraperitoneal injection 30 min before challenging.

### Determination of allergy

According to previous study [19], the allergy symptoms were evaluated every 5 min after sensitization. The symptom score scale for horse serum-induced anaphylactic shock and the rectal temperature were measured. The mild allergy was characterized by a ≤ 2 °C reduction in rectal temperature. The severe allergy was characterized by a shock state in which the rectal temperature was reduced by ≥ 5 °C (35.6–30.1 °C). Symptoms were observed within 60 min after sensitization and the severity of sensitivity was classified as mild, moderate, or severe. Mice were sacrificed at 60 min after sensitization.

### Sample preparation

Mice were euthanized by CO_2_ asphyxiation, and treated by cervical dislocation. The blood was collected, and then treated with anticoagulant coagulant for 4 h. After centrifuging at 352 × g for 15 min, the supernatant was obtained, and stored at −80 °C for use. The *E. granulosus* infection was observed by laparotomy. The lymph nodes from the neck, armpit, and groin were collected, and then placed in RPMI 1640 (Life Technologies Inc., CA, USA) for flow cytometry. At the same time, half of the left lung was collected for immunohistochemistry.

### Flow cytometry

The immune cells isolated from the mouse lymph nodes were analyzed by flow cytometry. The lymph node cells were harvested from all three groups. The cell suspensions were prepared in complete RPMI 1640. The nonspecific binding sites were blocked for 30 min at 4 °C using ice-cold phosphate-buffered saline supplemented with 1 % normal rat serum. After extensive washing, the cells were stained using CD4 + CD25 + FOXP3+ antibodies according to the protocol provided with the Mouse Regulatory T Cell Staining Kit (eBioscience Inc., CA, USA). The stained cells were analyzed by using a FACS-LSRII flow cytometer (BD Biosciences Inc., CA, USA). The data were analyzed using FCS Express (De Novo Software; De Novo Inc., CA, USA).

To evaluate the effectiveness of DEX, regulatory T cells in experimental allergic mice and the percentage of CD4 + CD25 + FOXP3+ regulatory T cells among the CD4+ T cells were analyzed in the lymph nodes of mice from all groups according to the manufacturer’s protocol (BD Biosciences Inc., CA, USA). The prepared cells were stained with anti-mouse CD4 FITC and anti-mouse CD25 APC, then fixed and permeabilized using the Foxp3 Staining Buffer Set (eBioscience Inc., CA, USA), and subsequently stained with 0.5 g of anti-mouse Foxp3 PE (Shanghai Sangon Biological Engineering Co., Ltd., Shanghai, China). Cells in the lymphocyte gate were analyzed using a FACSCalibur cytometer equipped with CellQuest (BD Biosciences Inc., CA, USA).

### Measurement of IgE, IgG, IgG1 and cytokines

Blood samples were taken from the angular vein in eyeball of mice, and then quickly placed in anticoagulant tubes, followed by storing at room temperature for 2 h. The samples were centrifuged at 352 x g for 10 min, and then the levels of IgE, IgG, IgG1 and cytokines (IL-10, IL-13, and TGF-β1) in serum were quantified by enzyme-linked immunosorbent assay (Eb Inc., Georgia, USA) according to the manufacturer’s instruction. The optical density was read at 450 nm.

### Immunohistochemistry

The 2-μm thick paraffin sections of left lung were prepared. The sections were deparaffinized, and then washed with distilled water. After soaking in PBS for 5 min, they were incubated with 3 % H_2_O_2_ at room temperature for 15 min. After washing with PBS, the sections were incubated with antibodies specific to histamine (1: 500; Sigma-Aldrich Corp., MO, USA) at 4 °C overnight, followed by washing with PBS. Then the sections were subsequently incubated with secondary antibody at 37 °C for 30 min. After washing with PBS, the DAB staining was performed, followed by flushing with water, restaining, dehydration and mounting. The sections were observed in Q550CW image acquisition and analysis system (Leica Science Lab, Berlin, Germany). Positive areas of histamine expression, as indicated by the detection of brown granules, were selected. Three positive areas were randomly selected from each section and the integrated optical density was calculated.

### Statistical analysis

All statistical analysis was carried out using SPSS17.0 software (SPSS Inc., Chicago, IL, USA). Data were presented as mean ± SD. Differences between the groups were assessed using a one-way ANOVA test with a post-hoc Bonferroni test for pairwise comparisons if the data followed a normal distribution or the Kruskal–Wallis test with a Mann–Whitney *U* test for pairwise comparisons if the data did not follow a normal distribution. *P* < 0.05 was considered as statistically significant.

## Results

### Model of CE infection and sensitization

The mean body weight was similar in all three groups (*P* = 0.302) (Table 1). CE-infected model were confirmed by abdominal ultrasonography and laparotomy. Only 20 of the 32 (62.5 %) vaccinated mice were successfully infected. Several botryoid cysts were found in each mouse with a minimum diameter of 1 mm and a maximum diameter of 3 mm. 16 CE-infected mice were randomized into untreated group and DEX group (n = 8 in each group).

**Table 1:** The condition of establishment of animal model

Variables	Uninfected group (n = 8)	Untreated group (n = 8)	DEX group (n = 8)	*p*-value
Body weight	23.61 ± 0.83	23.05 ± 0.56	23.25 ± 0.73	0.302
Severity of allergy, score				
None	0 (0 %)	0 (0 %)	1(12.5 %)	
Mild	0 (0 %)	5 (62.5 %)	5 (62.5 %)	
Moderate	0 (0 %)	0 (0 %)	1 (12.5 %)	
Severe	0 (0 %)	3 (37.5 %)	1 (12.5 %)	
ND	8(100 %)	0 (0 %)	0 (0 %)	

Data were presented as mean ± SD for body weight and n (%) for severity of allergy. Differences among groups were compared using one-way ANOVA test for age ND, not done

**Fig. 1:**
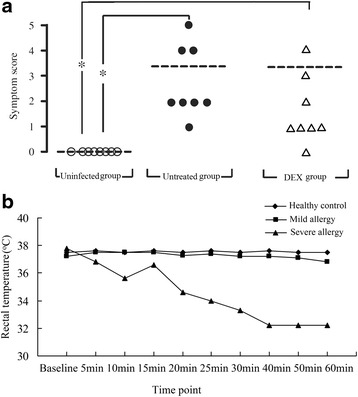
Induction of anaphylaxis in untreated and DEX treated mice. Mice inoculated with protoscoleces from *E. granulosus* and sensitized using a cyst fluid suspension to induce anaphylactic shock. **a**, Peak anaphylactic symptom score of each mouse in different group within 60 min after challenge. **b**, The different changes of rectal temperature with time in different severity of allergy

**Fig. 2:**
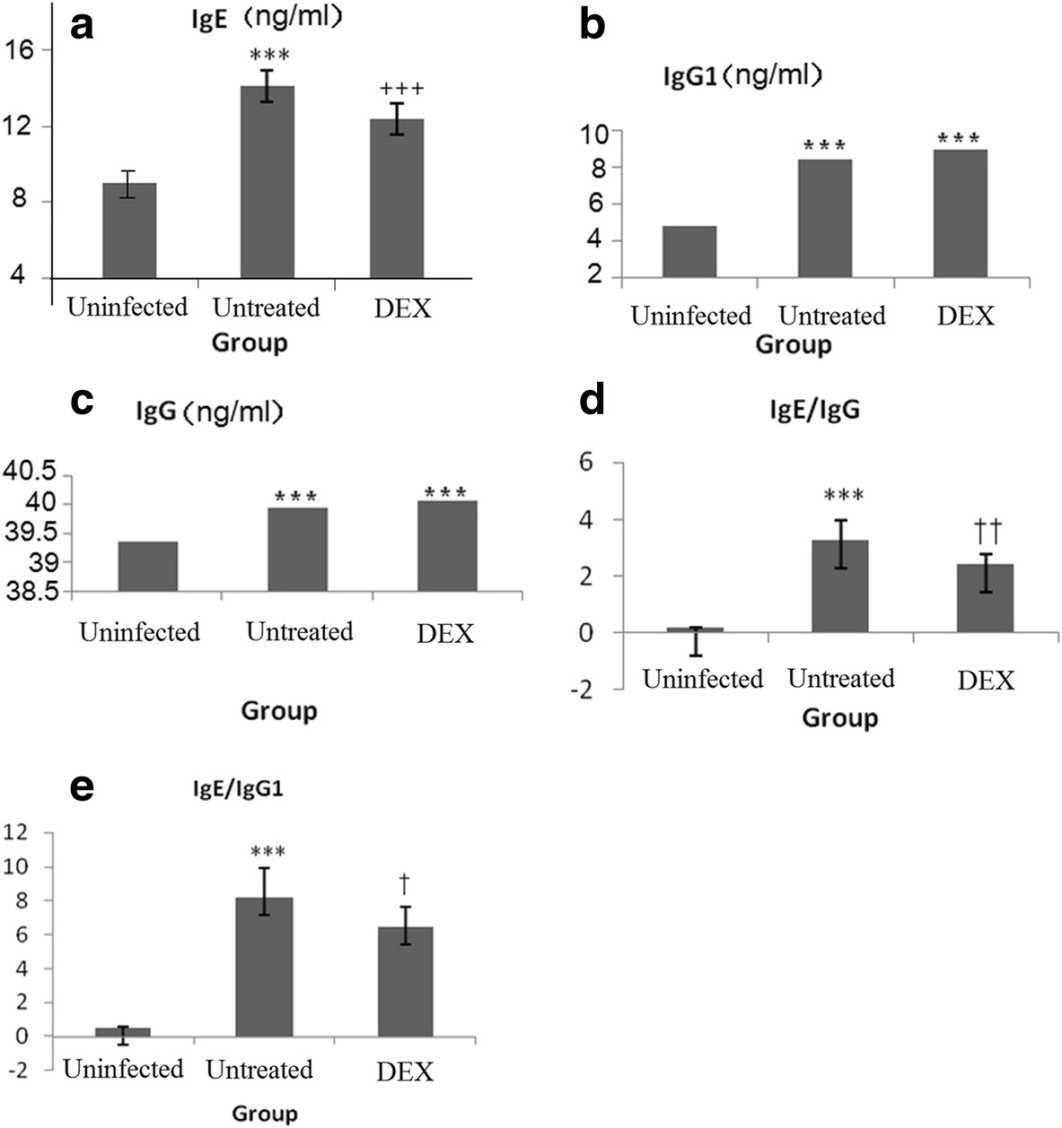
The results of antigen specific antibody in serum, IgE (**a**), IgG1 (b), IgG (**c**), ratio of IgE/IgG (**d**), and ratio of IgE/IgG1 (**e**) by group. ^*, †^ *P* < 0.05, ^**, ††^ *P* < 0.01,^***, †††^ *P* < 0.001, indicate significantly different as compared with group A^*^ and group B^†^, respectively

**Fig. 3:**
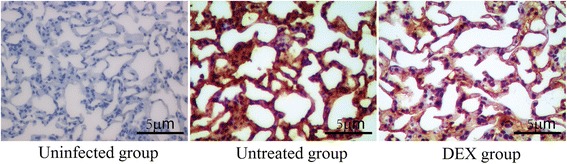
Detection of lung histamine by immunohistochemistry

Allergy score and rectal temperature were used to determine the linical symptoms of sensitization (Fig. 1), as well as the levels of lung histamine and serum IgE (Figs. 2 and 3). It was found that 93.75 % of all sensitized mice developed allergic symptoms. Only one mouse in DEX group showed no symptoms of allergy, five presented with mild allergy, one with moderate allergy, and one with severe allergy. In untreated group, five mice exhibited mild allergy and three exhibited severe allergy; the allergy severity for uninfected group was not assessed.

The clinical symptoms after challenge were shown in Fig. 1a. Most mice starting with continuous mouth-scratching and ear canal digging with hind legs (score 1/5), and the symptoms rapidly progressed to puffiness around eyes and/or mouth and self-isolation (score 2/5). The most severe reactions provoked no response to prodding, tremor, and death. The kinetics and severity of the anaphylactic reactions were consistent among mice.

The changes in rectal temperature with time with severity of the allergic reaction were shown in Fig. 1b. The rectal temperature in the uninfected group remained stable. The mice with a mild allergic reaction experienced a small variation in rectal temperature of less than 2.0 °C; however, the variation in the rectal temperature in the severe allergy mice was larger at more than 5 °C. In most of the mice, the rectal temperature began to change 5 min after sensitization. Anaphylactic shock occurred approximately 40 min later and the mice were then sacrificed.

Figure 2 presented the levels of antigen-specific antibody in serum. After infected,the levels of IgE, IgG, and IgG1 were significantly higher (*P* < 0.001). However, the IgE level was significantly lower in DEX group than that in untreated group (*P* < 0.001) (Fig. 2a–c). The ratio of IgE/IgG and IgE/IgG1 displayed a similar trend compared to that observed with the IgE level (Fig. 2d, e).

### Histamine levels

Pulmonary histamine levels were determined by immunohistochemical analysis and representative images are shown in Fig. 3a–c. Mice in uninfected group expressed almost no histamine, but histamine expression increased after sensitization. In the DEX group, histamine expression was reduced compared to that in uninfected group. The results revealed that treatment with DEX almost eliminated the inflammatory infiltrate in mice (Fig. 3c).

### Detection of lymphocyte populations and serum cytokine levels

**Fig. 4:**
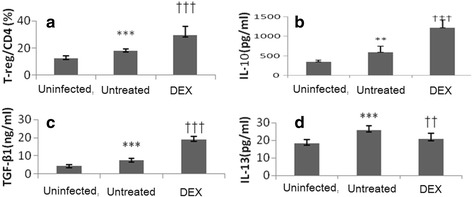
In mice peripheral lymph nodes, % of CD4 + CD25+ FOXP3+ (Treg cells) (**a**), and Data were presented as mean ± SD by group. ^***, †††^ *P* < 0.001, indicate significantly different as compared with group **a** ^*^ and group **b** ^†^, respectively. Presents the level of TGF-β1, IL-10, and IL-13, in serum by group. The results showed that DEX group had significantly higher level of TGF-β1 and IL-10 than uninfected group and untreated group (All *P* < 0.001) (**b**, **c**). The IL-13 level was significantly higher in untreated group B than the other two groups (both *P* < 0.001, **b**)

Figure 4 presented the proportion of CD4 + CD25 + FOXP3+ Treg cells relative to CD4+ Treg cells in the peripheral lymph nodes and the levels of IL-10, TGF-β1, and IL-13 in serum at each group. Treg cells were assessed by flow cytometry, as shown in Fig. 4a. Cytokines, including IL-10 and TGF-β, are crucial for the maintenance of Treg cells as well as their function and Foxp3 expression, as shown in Figs. 4b and c. IL-13 on behalf of Th2 cytokines is shown in Fig. 4d.

The results revealed that the percent of CD4 + CD25 + FOXP3+ Treg cells relative to CD4+ cells was significantly higher in DEX group than those in uninfected group and untreated group (uninfected group: 12.51 ± 1.68; untreated group: 17.92 ± 1.29; DEX group: 29.3 ± 6.54; both *P* < 0.0001) (Fig. 4a). The results showed that DEX group had significantly higher levels of IL-10 and TGF-β1 than those in uninfected group and untreated group (all *P* < 0.001) (Fig. 4b, c). The IL-13 level was significantly higher in untreated group than in other groups (both *P* < 0.001) (Fig. 4d).

**Fig. 5:**
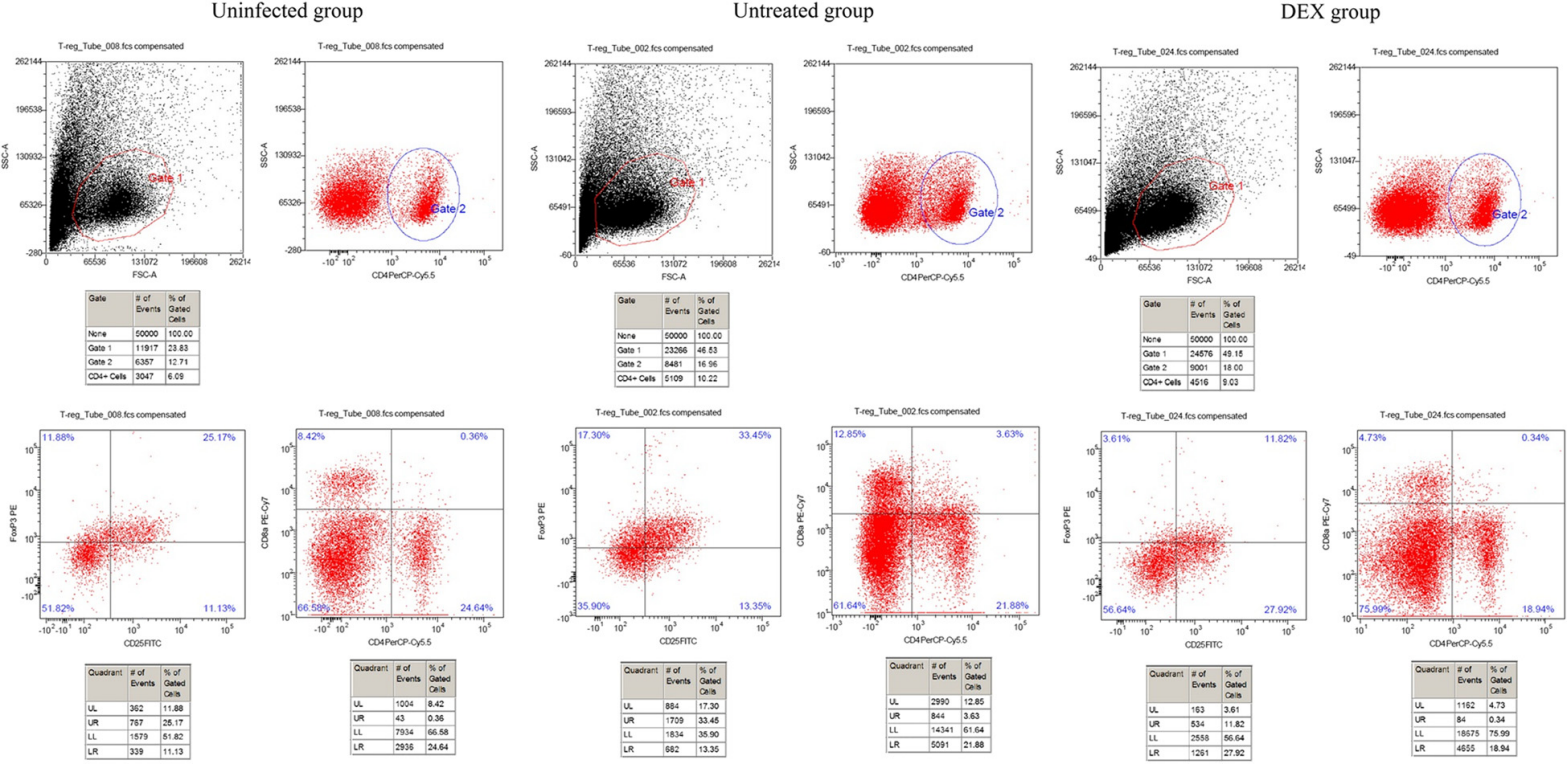
Results of flow cytometry. The percentage of CD4 + CD25 + FOXP3+ regulatory T cells among CD4+ T cells in peripheral lymph nodes of experimental mice in 3 groups

As shown in Fig. 5, the percentage of CD4 + CD25 + FOXP3+ Treg cells among CD4+ cells in the peripheral lymph nodes in the DEX group was higher than the percentages in the other groups.

## Discussion

This study investigated the effect of DEX in CE-induced allergic reactions, especially the role of CD4 + CD25 + FOXP3+ Treg cells and some cytokines. C57BL/6 mice were firstly inoculated with *E. granulosus* to set up CE-infected model, then some mice were sensitized with cystic fluid to set up challenge model. DEX has been preliminary used. After sensitization, the sensitized CE-infected mice had higher serum IgE levels and lung histamine expression than that observed in the DEX group (*P* < 0.001). Furthermore, the sensitized mice had significantly higher IgE/IgG and IgE/IgG1 levels as compared to those observed in uninfected group and the DEX group. Moreover, in terms of clinical symptoms, the severity of allergy scores suggests that only 12.5 % of the mice in the DEX group had a severe allergic reaction; however, in untreated group, the proportion was as high as 37.5 %. Treatment with DEX can reduce the severity of allergic reactions induced by CE.

In addition, the proportion of CD4 + CD25 + FOXP3+ Treg cells relative to CD4+ Treg cells was significantly higher in the DEX group (*P* < 0.001); significantly higher IL-10 and TGF-β1 levels were also noted in this group (all *P* < 0.001). In contrast, the IL-13 levels were significantly higher in the sensitized mice alone (both *P* < 0.001). Thus, our study confirmed that the levels of TGF-β1 and IL-10, as well as the proportion of CD4 + CD25 + FOXP3+ Treg cells relative to CD4+ Treg cells, exhibited a similar tendency: they all decreased after sensitization, but increased after treatment with DEX.

The T-cell network is considered central to the balance and prevention of Th1 and/or Th2 diseases. Research has shown that the proportion of CD4 + CD25 + FOXP3+ Treg cells relative to CD4+ Treg cells is very important in allergic disorders and parasitic invasions. *Schistosoma mansoni* infection has been associated with protection against allergies [20]. The mechanisms underlying this association may involve regulatory cells and cytokines. Our research found that the proportion of CD4 + CD25 + FOXP3+ Treg cells relative to CD4+ Treg cells increased after *E. granulosus* infection was consistent with previous studies [21] and significantly increased with preliminary treatment with DEX, which suggested their possible role to contributed to the maintenance of immune tolerance. However, the proportion of Treg cells relative to CD4+ cells was reduced in untreaded mice when allergic reaction happened,just as previously described [22]. In the pollen season, the sensitization patients have lower number of Treg cells than healthy people [23]. The reduction of Treg cells may induce the occurrence of asthma; Because DEX, which has a role in the allergic response, interacts with Treg cells in many immune reactions and has an effect on the allergic reaction [24] in mouse models of OVA-induced asthma [21, 25], further studies will analyze their role in sensitization in this model.

Just as proved in previous studies [26–28], Treg cells may exert their effects via IL-10 and TGF-β which are crucial for the maintenance and function of Treg cells. Following preliminary treatment with DEX, our research showed increased level of Treg cells consistent with the greater serum levels of both IL-10 and TGF-β1, which were significantly reduced after sensitization. IL-10 may exert anti-allergic effects via inhibiting macrophage activation and cytotoxicity, and IL-10 is crucial for maintain immune tolerance for the better of parasites survival and growth [29]. The increased TGF-β1 levels after treatment with DEX are consistent with the results of previous studies [21, 24, 30].

Although the proportion of Treg cells and the serum levels of IL-10 and TGF-β1 were reduced after sensitization, the level of IL-13 increased. It is shown in previous studies [31, 32] that cystic fluid-induced sensitization is accompanied by increased levels of Th2 cells, which is characterized by increased IL-13 levels. The allergic reaction is associated with high levels of IgE and IL-13. Treg cells can suppress the allergic reaction directly through inhibit Th2 cells [33]. DEX may maintain the immune tolerance, increasing the levels of protective IL-10 and markedly reducing levels of IL-13. The effect of DEX may be related to the increased FOXP3 expression [34]. Further studies will assess the effects of it.

There were limitations in the present study. First, the composition of the allergens is complex, the exact factors in the roughly prepared cystic fluid, which mediate the sensitization, are not investigated further. We did not use standard allergen, such as ovalbumin [35], to induce the allergic reaction. Furthermore, we have only studied the effect of DEX, but there are many other kinds of glucocorticoids drugs, e.g., hydrocortisone, which need further researches. In addition, although the effect of DEX in allergy in CE shock is investigated, the mechanism by which the sensitization reduces the Treg cells and cytokine levels has not been explored, so further study is needed.

## Conclusion

In conclusion, DEX has protective effect on CE-induced allergic reaction by adjusting Treg cells. This protective effect may be due to Treg cells upregulating IL-10 and TGF-β1 levels, and inhibiting helper T cell 2 cytokines. Further studies should be performed to elucidate the intrinsic mechanism underlying the drug treatment in CE-induced anaphylactic reaction.

## Abbreviations

CE: cystic echinococcosis
CE: cystic echinococcosis
DEX: dexamethasone
EU: endotoxin units
IL-10: interleukin-10
IL-13: interleukin-13
LPS: lipopolysaccharide
SD: standard deviation
SHF: sheep hydatid fluid
T cells: treg cells
TGF-β1: tumor growth factor-β1
Th1: helper T cell 1
Th2: helper T cell 2
